# Small granule protein CP2 of *Cryptosporidium* translocates to the parasite-host interface during invasion and localizes to parasitophorous vacuole membrane in association with other secretory proteins

**DOI:** 10.1371/journal.ppat.1013847

**Published:** 2025-12-31

**Authors:** Fuxian Yang, Yujin Huang, Ping Zhu, Shengchen Zhang, Jilei Huang, Haoyu Chen, Xiaoqing Gong, Yaqiong Guo, Na Li, Rui Xu, Yaoyu Feng, Lihua Xiao

**Affiliations:** 1 State Key Laboratory of Animal Disease Control and Prevention, Center for Emerging and Zoonotic Diseases, College of Veterinary Medicine, South China Agricultural University, Guangzhou, China; 2 The Instrumental Analysis & Research Center, South China Agricultural University, Guangzhou, China; The Walter and Eliza Hall Institute of Medical Research, AUSTRALIA

## Abstract

*Cryptosporidium* is a major cause of diarrhea in humans and farm animals for which there are no effective drugs. The parasite resides in a parasitophorous vacuole on the surface of gastrointestinal epithelial cells. Although the parasitophorous vacuole membrane (PVM) plays a critical role in parasite development and survival, its composition and biogenesis are not well understood. In this study, we used reverse genetic tools to investigate the secretion and function of a potential PVM protein called CP2. Endogenous gene tagging revealed that, following biosynthesis, CP2 is stored in the small granules (SG) within sporozoites. Upon invasion of host cells, CP2 is translocated to the parasite-host interface. During intracellular development, CP2 is distributed across the entire PVM in trophozoites and microgamonts, but it is concentrated in the lower PVM, near the attachment zone, in mature meronts and macrogametes. Interestingly, CP2 is dispensable; deleting the *CP2* gene did not significantly affect the growth or pathogenicity of a virulent *Cryptosporidium* strain. *CP2* knockout resulted in increased expression of a neighboring gene encoding SG3, which is also translocated from the SG to the PVM. SG3 was further localized to knob-like and filamentous structures outside the PVM. Together, these findings suggest that CP2 is secreted to the parasite-host cell interface during invasion and intracellular growth, where it potentially contributes to the formation of the nascent parasitophorous vacuole of *Cryptosporidium*, together with other SG proteins.

## Author summary

The enteric parasite *Cryptosporidium* survives inside host cells by forming a protective compartment known as the parasitophorous vacuole. This structure is essential for establishing an infection, yet the process by which it forms remains unknown. We discovered that CP2, a well-known *Cryptosporidium* glycoprotein, is secreted from the small granules (SG) to the parasite-host interface during invasion, where it becomes is part of the nascent parasitophorous vacuole. Interestingly, the parasite can survive withoutCP2, possibly by increasing the production of another SG protein, SG3. This finding reveals an unexpected flexibility in the parasite’s infection strategy. We also identified two SG3-positive structures that may potentially facilitate interaction between parasites and host cells. These findings shed light on the mechanisms of *Cryptosporidium* infection and highlight novel aspects of its biology.

## Introduction

Cryptosporidiosis, a diarrheal disease caused by *Cryptosporidium* spp., is a significant global health problem affecting infants and neonatal animals [1]. It is also a leading cause of life-threatening diarrhea in immunocompromised individuals, particularly those with AIDS [2]. In low- and middle-income countries, pediatric cryptosporidiosis is associated with malnutrition, stunted growth, and delayed cognitive development [3]. In the United States, Europe, and other high-income countries, cryptosporidiosis is the most commonly identified cause of waterborne outbreaks of diarrhea [4]. Currently, treatment options for cryptosporidiosis are limited. The only approved treatment drug, nitazoxanide, has been shown to have low efficacy in malnourished children and patients with AIDS [5]. Of the nearly 50 known *Cryptosporidium* species, *C. hominis* and *C. parvum* are responsible for the majority of human infections [6].

*Cryptosporidium* completes its complex development within a single host. After a host ingests oocysts, the sporozoites released attach to the epithelial cells lining the gastrointestinal tract. This process induces the remodeling of the host cytoskeleton, including the recruitment of F-actin to the attachment sites, as well as modification of the host cell membrane and microvilli surrounding the parasites [7]. This ultimately leads to the formation of a parasitophorous vacuole (PV), which envelops the invading sporozoite at the tip of the epithelial cells. A complex structure also forms at the host-parasite interface: the feeder organelle (FO) [8]. The PV is intracellular but extracytoplasmic, distinguishing *Cryptosporidium* from most other apicomplexans [9]. However, the molecular composition and biogenesis of the PV membrane (PVM) remain largely unknown.

*Cryptosporidium,* like other apicomplexans, possesses secretory organelles such as rhoptries and dense granules (DG) that are critical for parasite invasion and PVM establishment [10]. PVM proteins have been extensively studied in *Toxoplasma gondii* because they are essential for parasite invasion, development, and virulence [11]. *Toxoplasma* rhoptry proteins, such as ROP1, ROP5, ROP17, and ROP18, are localized to the host cytosolic side of the PVM and play an important role in immune evasion [12]. Similarly, numerous DG proteins (known as GRA in *T. gondii*) have been identified in the PVM. Among them, GRA15 has been shown to regulate of NF-κB gene expression [13], and GRA7 has been shown to cooperate with ROP18 in neutralizing immunity-related GTPases [14]. Recently, several rhoptry proteins and DG proteins have been found in the PV of *C. parvum*, including ROP2, ROP4, ROP7, and DG4 [7,10]. Other proteins associated the *C. parvum* PVM include the surface protein CP2 [15], fatty acyl-CoA binding protein [16], lactate dehydrogenase [17], and fatty acyl-CoA synthetase isoforms [18]. However, the precise subcellular localizations and functions of these proteins remain unclear. Additionally, a novel secretory organelle, the small granule (SG), has been identified near the nucleus of *C. parvum* sporozoites. One SG protein, SG1, has been shown to be secreted to the parasite-host cell interface after invasion, while another SG protein, SG2, is localized in the PV [10].

In this study, we demonstrate that CP2 is a highly expressed SG protein in *C. parvum*. During host cell invasion, CP2 is translocated to the apical end of the parasite and secreted to the parasite-host interface. Once the PV is established, CP2 localizes to the PVM near the parasite-host interface. Translocation and secretion of CP2 can be blocked by deleting its signal peptide. Knocking out the *CP2* gene does not significantly affect the growth or pathogenicity of a virulent *C. parvum* strain, but appears to upregulate the expression of a neighboring gene that encodes another SG protein: SG3. SG3 is also translocated to the PVM alongside with two DG proteins and is further localized to SG3-positive knob-like and filamentous structures that outside the PVM. Together, these results suggest that both DG and SG proteins are integral components of the PVM and therefore potentially contribute to forming the intracellular niche.

## Results

### CP2 is a small granule protein of *Cryptosporidium*

**Fig 1:**
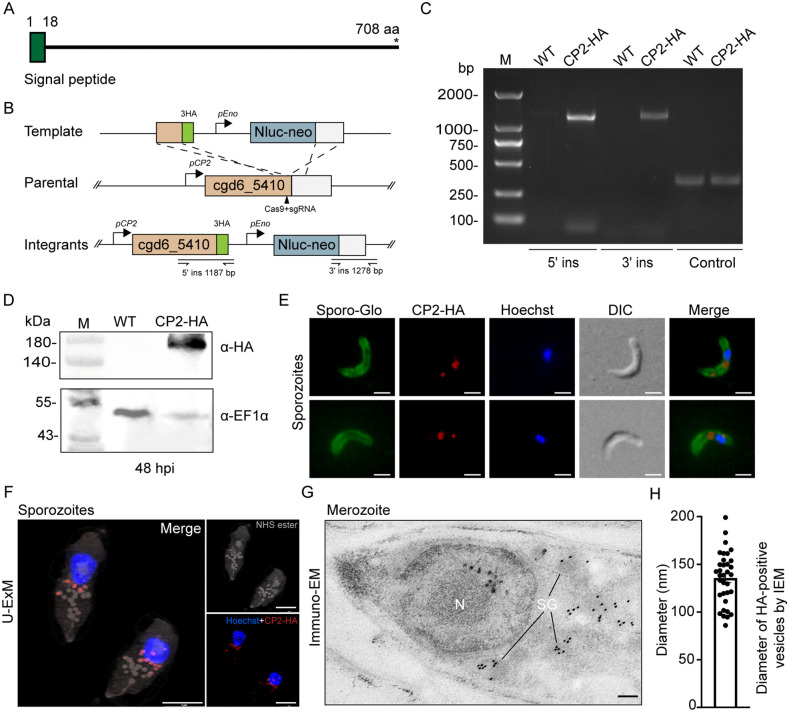
CP2 is expressed in small granules of *Cryptosporidium parvum.* **(A)** Schematic of the domain structure of CP2, including the putative signal peptide in green. Numbers indicate the residues from 1 to 708. **(B)** Schematic of the endogenous tagging of the *CP2* gene with a 3HA tag using CRISPR/Cas9. A single guide RNA (sgRNA) targeting the C-terminus of the *CP2* gene, the repair template for homologous recombination, and primers use in diagnostic PCR are shown. Nluc, nanoluciferase; neo, neomycin resistant marker; *pEno*, enolase promoter; *pCP2*, CP2 promoter. **(C)** Confirmation of the integration of the repair template in the CP2-HA line by PCR analysis of genomic DNA extracted from wild-type (WT) and CP2-HA parasites. The locations of primers used to verify 5’ integrations (5’ ins) and 3’ integrations (3’ ins) are indicated in (B), and an irrelevant gene (cgd2_920) was used as the DNA control. **(D)** Verification of the CP2 tagging by Western blot analysis of lysates of HCT-8 cultures infected with the wild-type (WT) and CP2-HA, using anti-HA and anti-EF1α antibodies. **(E)** Immunofluorescence localization of CP2-HA in sporozoites using rabbit anti-HA (red), Sporo-Glo that recognizes the whole parasite (green), and Hoechst (blue). Scale bars, 2 μm. **(F)** Ultrastructure expansion microscopy (U-ExM) of the CP2-HA expression in sporozoites. CP2-HA sporozoites were fixed, expended in water-based gel, and stained with rat anti-HA (red), NHS ester that recognizes granules (grey), and Hoechst (blue). Scale bars, 5 μm. **(G)** Ultrastructural localization of CP2-HA in merozoites by immunoelectron microscopy (immuno-EM) of the ileal tissue from mice infected with CP2-HA, using rabbit anti-HA and 10-nm colloidal gold-conjugated goat-anti-rabbit IgG. N, nucleus; SG, small granules. Scale bars, 100 nm. **(H)** Size of CP2-HA-positive vesicles in the immuno-EM of CP2-HA merozoites.

The CP2 protein, encoded by the cgd6_5410 gene [15], was predicted to be an 80 kDa protein containing a signal peptide (Fig 1A). Further analysis revealed that CP2 is an intrinsically disordered protein with no known functional domains, and it is a threonine/serine-rich secretory protein (S1A and S1B Fig). To localize CP2 expression in *C. parvum*, three copies of the hemagglutinin epitope (3 × HA) and a Nluc-neo selection cassette were fused to the C-terminus of the *CP2* gene (Fig 1B). After infecting GKO mice with sporozoites transfected with the Cas9 and replacement plasmids and selecting transgenic parasites with paromomycin, a *C. parvum* line (CP2-HA) in which the *CP2* gene was tagged with 3 × HA was obtained. The correct genomic insertion was confirmed by diagnostic PCR using primers flanking the inserted cassette (Fig 1B and 1C). We analyzed the expression of CP2 in HCT-8 cultures infected with CP2-HA and WT for 48 h using Western blot and a monoclonal antibody against HA. A band of ~160 kDa was identified in the CP2-HA sample but not in the WT sample, confirming normal CP2 expression after tagging (Fig 1D). However, the size of CP2 in the Western blot was larger than predicted. This could be due to the putative O-glycosylated nature of the protein (S1B Fig) and the high occurrence of positively charged amino acids (almost 20%) and low occurrence of hydrophobic amino acids (only 30%) (S1C Fig). They likely resulted in the slower migration of the naive CP2 in SDS-PAGE.

In IFA of sporozoites, CP2-HA expression was detected mainly in the vicinity of the nucleus (Fig 1E). To further identify the subcellular localization of CP2, we used ultrastructure expansion microscopy (U-ExM) with immunofluorescence staining of CP2-HA with anti-HA antibody and N-hydroxy succinimide (NHS) ester staining of the rhoptry, dense granules (DG), small granules (SG) and micronemes in sporozoites [10]. CP2-HA expression was detected in numerous SGs around the nucleus (Fig 1F), consistent with the localization of SG1, SG2, and SKSR1 in recent studies [10,19]. The SG localization of CP2-HA was also confirmed by IEM, which showed the presence of gold particles in the SG around the nucleus of merozoites (Figs 1G and S2), with the diameter of HA-positive granules being 135 ± 27.03 nm (Fig 1H). Previous studies have shown that the SGs are close to the parasite nucleus and are smaller in diameter than the DGs, which are located more anteriorly and approximately 200 nm in size [10]. Taken together, these observations suggest that CP2 is a small granule protein in *C. parvum*.

### CP2 is secreted to the parasite-host interface during invasion and translocates to the PVM after parasite adhesion

**Fig 2:**
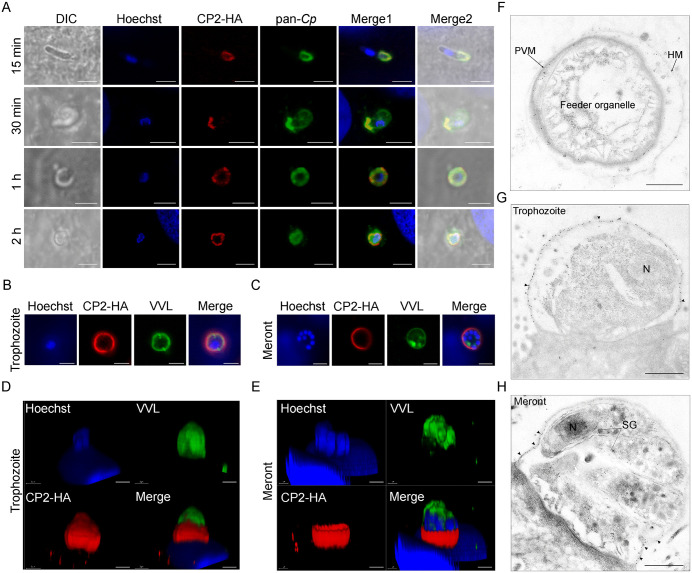
CP2 localizes to the parasitophorous vacuole in intracellular stages of *Cryptosporidium parvum.* **(A)** Dynamics of CP2-HA secretion during invasion and early growth of *C. parvum* as revealed by immunofluorescence microscopy. HCT-8 cells were infected with CP2-HA sporozoites, fixed at 15 min, 30 min, 1 h and 2 h post-infection (hpi), and stained with rabbit anti-HA (red), anti-*Cp* that recognizes whole parasites (green), and Hoechst (blue). Scale bars, 2 μm. **(B-E)** Immunofluorescence localization of CP2-HA in trophozoites and meronts in HCT-8 cultures. HCT-8 cells were fixed at 24 hpi and stained with rabbit anti-HA (red), *Vicia villosa* lectin (VVL, green) and Hoechst (blue). Both the top (B and C) and side (D and E) views of the same parasites are shown. Scale bars, 2 μm. **(F-G)** Ultrastructural localization of CP2-HA in the feeder organelle (F), trophozoite (G), and meront (H) by immunoelectron microscopy of the ileal tissue from mice infected with CP2-HA, using a rabbit anti-HA antibody and 10-nm colloidal gold-conjugated goat-anti-rabbit IgG. Black arrowheads indicate the distribution of gold particles. N, nucleus; SG, small granules. Scale bars, 500 nm.

To identify the intracellular localization of CP2, we tracked CP2 expression at different stages of infection with the CP2-HA line. During early invasion, CP2 moved together with the SG from the middle to the apical end of the sporozoite (Fig 2A, top panel). Soon after, CP2 formed a ring-like structure at the interface between the sporozoite and the host cell (Fig 2A). In young trophozoites and late developmental stages such as immature and mature meronts and macrogametes, CP2 partially colocalized with the *Vicia villosa* lectin (VVL), which mainly stains the PVM (S3A Fig). Furthermore, based on Z-stack rendered 3D images in confocal microscopy, CP2 was found over most of the surface of trophozoites and mainly at the base of meronts (Fig 2B–2E). To further identify the subcellular localization of CP2, we performed IEM on ileal tissue from mice infected with the CP2-HA line. CP2 was observed in the PVM but not in the feeder organelle (FO) (Fig 2F). Furthermore, the localization of CP2 was dynamic and changed during parasite development. In trophozoites and microgamonts, CP2 accumulated throughout the PVM (Figs 2G and S3B, S3C). In contrast, in meronts and macrogametes, CP2 was expressed only at the base of the PVM above the FO (Figs 2H and S3D). Taken together, CP2 is secreted into the parasite-host interface during invasion and localizes to the PVM of developmental stages.

To investigate the necessity of the signal peptide for CP2 translocation, we deleted the signal peptide (amino acids 1–18) and added a 3HA tag to the C-terminus of CP2 using CRISPR/Cas9 (S4A Fig). Diagnostic PCR confirmed the correct integration of the replacement cassette into the genome after selecting and amplifying the transgenic parasites in GKO mice (S4B Fig). IFA of sporozoites revealed that *CP2ΔSP* remained near the parasite nucleus (S4C Fig). However, in intracellular stages, *CP2ΔSP* was found in the cytosol rather than in the PVM (S4D Fig). These results suggest that the signal peptide is necessary for CP2 translocation and secretion during invasion.

### Knocking out the *CP2* gene does not significantly affect the growth and pathogenicity of a virulent *C. parvum* strain

**Fig 3:**
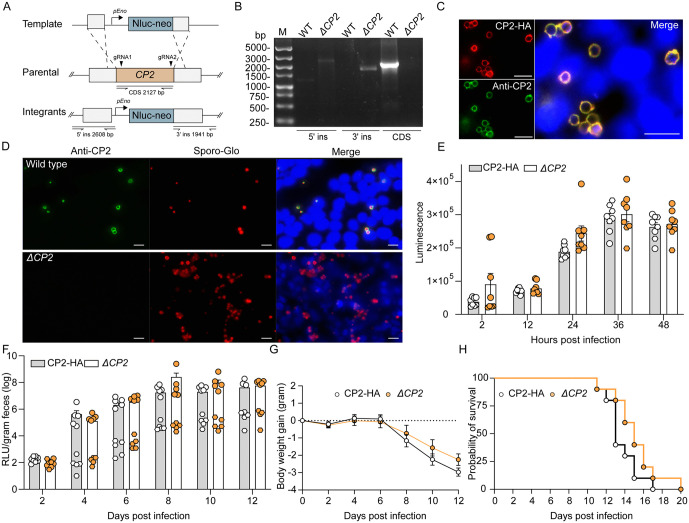
CP2 is dispensable in *Cryptosporidium parvum.* **(A)** Schematic for knocking out the *CP2* gene in *C. parvum*. The *CP2* gene was replaced by an Nluc-Neo cassette via the CRISPR/Cas9 method. The locations of the two gRNAs used are shown. Nluc, nanoluciferase; neo, neomycin resistance marker; *pEno*, enolase promoter. **(B)** Verification of the integration of the repair template in the *ΔCP2* line by PCR analysis of DNA extracted from wild-type (WT) and *ΔCP2* parasites. The locations of primers used to verify 5’ ins, 3’ ins, and CDS of the *CP2* gene are indicated in (A). **(C)** Immunofluorescence analysis (IFA) of CP2 expression in *C. parvum* in HCT-8 cells infected with CP2-HA for 24 h, using rabbit anti-HA antibodies (red), anti-CP2 antibodies (green), and Hoechst (blue). Scale bars, 10 μm. **(D)** Verification of *CP2* deletion in the *ΔCP2* line by IFA of HCT-8 cultures infected with wild-type or *ΔCP2* parasites for 48 h, using anti-CP2 antibody (green), Sporo-Glo that recognizes whole parasite (red), and Hoechst (blue) Scale bars, 10 μm. **(E)** Comparison of the growth of CP2-HA and *ΔCP2* lines in HCT-8 cells as indicated by luminance levels at individual time points. Each bar represents the mean ± SEM of data from eight replicates in two independent experiments. The *P* values from a two-way ANOVA with Sidak’s multiple comparisons were all less than 0.05. **(F)** Parasite burden of GKO mice infected with 1 × 10^3^ CP2-HA or *ΔCP2* oocysts, as indicated by luciferase activity in fecal pellets. Each bar represents the mean ± SEM of data from ten GKO mice in two independent experiments. All mice in each infection experiment were housed separately from each other in individual cages (one mouse per cage). **(G and H)** Weight changes (mean ± SEM, n = 10) and survival curve of GKO mice infected with CP2-HA or *ΔCP2* during the infection experiments.

To better understand its role in *Cryptosporidium*, we created a transgenic line of the virulent IIdA20G1-HLJ strain lacking the *CP2* gene using CRISPR/Cas9 (Fig 3A). A *ΔCP2* line was easily obtained from GKO mice infected with the transgenic parasites. We confirmed the correct integration of the replacement cassette by diagnostic PCR (Fig 3B), and validated CP2 ablation by IFA using polyclonal antibodies against recombinant CP2 expressed in *E. coli* cells. IFA of CP2-HA parasites in HCT-8 cultures revealed that anti-CP2 antibodies exhibited ring-like staining that co-localized with the HA tag (Fig 3C). In contrast, no CP2 expression was detected in HCT-8 cultures infected with the *ΔCP2* line using anti-CP2 antibodies (Fig 3D), confirming the absence of protein expression following *CP2* gene deletion.

To evaluate the importance of CP2 in parasite growth, we infected HCT-8 cells with CP2-HA and *ΔCP2* parasites, and then compared the parasite load by measuring the luminescence of the transgenic parasites at different time points. In two infection studies, the CP2 deletion strain exhibited no significant growth defect *in vitro* (Fig 3E), suggesting that CP2 is not essential for parasite proliferation under these conditions. To investigate the role of CP2 in parasite virulence *in vivo*, we infected GKO mice with CP2-HA and *ΔCP2* oocysts and monitored the luminescence levels in feces from 0 dpi to 12 dpi. There was no significant difference in luminescence levels or body weight gain between GKO mice infected with CP2-HA and *ΔCP2* in two infection studies (Fig 3F and 3G). Additionally, CP2 ablation only slightly delayed the death of infected mice (Fig 3H).

### Knocking out *CP2* alters the transcription of two neighboring *C. parvum* genes

**Fig 4:**
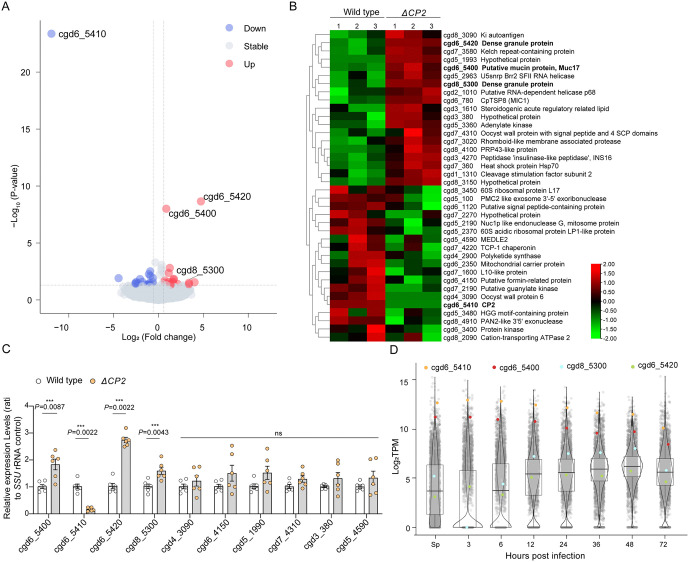
Deletion of the *CP2* gene leads to increased expression of neighboring genes. **(A)** Volcano plot of differentially expressed genes (DEGs) in an RNA-seq analysis of HCT-8 cells infected with WT or *ΔCP2* parasites for 48 h. Upregulated genes are shown in red, with a fold change of gene transcription level of ≥ 1.5 and a padj ≤ 0.05 in *ΔCP2* vs WT. Downregulated genes are shown in blue, with a fold change of gene transcription level of ≤ -1.5 and a padj ≤ 0.05. **(B)** Heatmap showing the 38 DEGs identified between wild-type (WT) and *ΔCP2* parasites in HCT-8 cultures. **(C)** Relative expression levels of the four downregulated genes (cgd6_5410, cgd4_3090, cgd6_4150, and cgd5_4590) and six upregulated gene (cgd6_5400, cgd6_5420, cgd8_5300, cgd5_1990, cgd7_4310, and cgd3_380) in qRT-PCR analysis of HCT-8 cultures infected with WT or *ΔCP2* parasites for 48 h. The small subunit (*SSU*) rRNA gene of *C. parvum* was used as an internal reference. The data are the mean ± SEM (n = 6 from two independent infection experiments). *P* values were determined using a two-tailed Mann-Whitney U test; ns, not significant. **(D)** Violin plots showing the relative expression levels of the *CP2* gene (cgd6_5410) and three significantly upregulated genes in sporozoites and developing stages of WT *C. parvum* in HCT-8 cells, as indicated by TPM values from RNA-seq analysis of the transcriptome [20].

To determine whether there were any compensatory changes in gene expression following the deletion of the *CP2* gene, we performed RNA-seq analysis on WT and *ΔCP2* parasites in HCT-8 cultures and searched for genes with at least a 1.5-fold difference in expression levels between the two groups. We identified 19 genes that were significantly upregulated and 19 genes that were significantly downregulated in *ΔCP2* parasites. The latter group included cgd6_5410 encoding CP2 (Fig 4A and 4B). The qRT-PCR results from HCT-8 cultures infected with WT and *ΔCP2* parasites for 48 h largely supported the upregulation of the cgd6_5400, cgd6_5420, and cgd8_5300 after *CP2* (cgd6_5410) deletion. As expected, the qRT-PCR analysis confirmed the absence of *CP2* expression in *ΔCP2* parasites. Six other genes that exhibited minor up- and downregulation in the RNA-seq analysis showed similar gene expression in the qRT-PCR analysis of HCT-8 cultures infected with WT and *ΔCP2* parasites (Fig 4C). Of the four genes with significant differences in gene expression, cgd6_5400 and cgd6_5420 are adjacent to the *CP2* gene (cgd6_5410), suggesting that there was a neighboring effect of the *CP2* deletion.

The proteins encoded by cgd6_5420 and cgd8_5300 were predicted to be DG proteins in a recent study [10]. RNA-seq analysis of the transcriptomes of *C. parvum* sporozoites and the developmental stages in HCT-8 cultures [20] revealed high transcript levels of cgd6_5400 (referred to as *SG3* below) and cgd6_5410 (*CP2*) at all stages. In contrast, we observed markedly lower transcript levels for two DG proteins encoded by cgd6_5420 (DG7) and cgd8_5300 (DG8) compared to CP2 (Fig 4D). This nomenclature aligns with the existing names DG5 and DG6 (encoded by cgd7_4490 and cgd7_4500, respectively) [21]. These results are consistent with the published single-cell atlas: SG3 and CP2 appear to be expressed throughout intracellular development, and the expression patterns of the DG7 and DG8 match those of other DG proteins [22]. Taken together, these results suggest that CP2 ablation alters the expression of several *C. parvum* genes, particularly the two neighboring genes.

### SG3, DG7, and DG8 also translocate to the PVM

**Fig 5:**
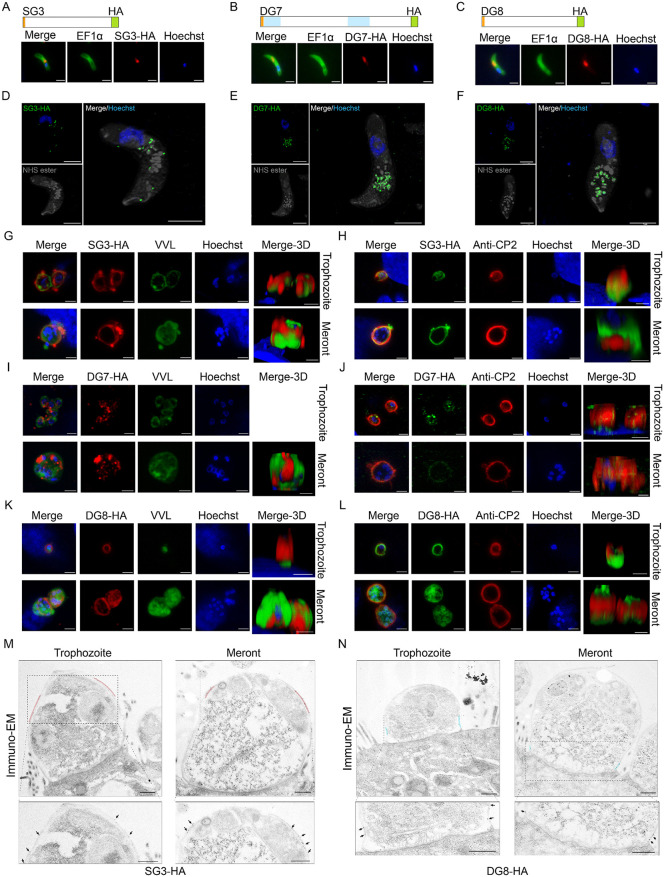
Characteristics of the expression of three novel PVM proteins of *Cryptosporidium parvum.* **(A-C)** Immunofluorescence localization of SG3-HA (cgd6_5400-HA), DG7-HA (cgd6_5420-HA), and DG8-HA (cgd8_5300-HA) in sporozoites. The schematics show the domain structure of SG3, DG7 and DG8, including the putative signal peptide (orange), predicted repeat domains (light blue), and the location of the 3HA tag. Sporozoites from the transgenic lines were fixed and stained with rabbit anti-HA (red), anti-EF1α (green), and Hoechst. Scale bars, 2 μm. **(D-F)** U-ExM of SG3-HA, DG7-HA and DG8-HA in sporozoites, after expansion in gel and staining with rat anti-HA (green), NHS ester (gey), and Hoechst (blue). Scale bars, 5 μm. **(G, I, and K)** Immunofluorescence localization of SG3, DG7 and DG8 in trophozoites and meronts in HCT-8 cells at 24 hpi, after staining with rabbit anti-HA (red), *Vicia villosa* lectin (VVL, green), and Hoechst (blue). Scale bars, 2 μm. **(H, J, and L)** Co-localization of SG3-HA, DG7-HA and DG8-HA with CP2 in trophozoites and meronts in HCT-8 cells at 24 hpi, after staining with rabbit anti-HA (green), anti-CP2 (red), and Hoechst (blue). Scale bars, 2 μm. **(M and N)** Ultrastructural localization of SG3-HA and DG8-HA in trophozoites and meronts by immunoelectron microscopy of the ileum from infected mice, using a rabbit anti-HA antibody and a 12-nm colloidal gold-conjugated goat-anti-rabbit IgG antibody. Scale bars, 200 nm.

We examined the expression of cgd6_5400, DG7 and DG8 by adding a 3 × HA tag to the C-termini of the proteins using CRISPR/Cas9 (Figs 5A–5C and S5A–S5C). Diagnostic PCR analysis of the transgenic lines confirmed the correct insertion of the HA tag into the three genes (S5D–S5F Fig). IFA with the anti-HA antibody revealed that cgd6_5400was expressed adjacent to the nucleus of sporozoites (Fig 5A), while DG7 and DG8 were detected in the anterior to mid-region of the sporozoites (Fig 5B and 5C). U-ExM results showed that cgd6_5400 was predominantly expressed in the SG (Fig 5D), while DG7 and DG8 were expressed in the DG as expected (Fig 5E and 5F). Furthermore, the localization of cgd6_5400 was similar to that of CP2 and partially colocalized with it in sporozoites and merozoites in U-ExM (S5G–S5I Fig). The subcellular localization of cgd6_5400, DG7 and DG8 was confirmed by IEM analysis of merozoites *in vivo*. For cgd6_5400, gold particles were observed in a small organelle that correspond to the shape and size of the SG (134 ± 24.70 nm in diameter; S5J and S5M Fig). Therefore, the protein encoded by cgd6_5400 is named as SG3, following the SG1 and SG2 [10]. In contrast, gold particles were observed in the canonical DGs for DG7 and DG8, which had a more elongated shape and a larger size (196 ± 32.55 nm and 246 ± 35.95 nm, respectively; S5K–S5M Fig).

To investigate the secretion of SG3, DG7 and DG8 during invasion and parasite development, we localized the expression of these proteins in intracellular stages using IFA with the anti-HA antibody. During invasion, both SG3-HA and DG8-HA were found to be localized to the apical end of the sporozoite and subsequently formed a circular pattern at the parasite-host interface (S6A and S6B Fig). Further, SG3-HA expression was detected in the PVM stained with VVL in both trophozoites and meronts (Fig 5G). It was primarily localized above CP2, which is located near the base of the PVM. Thus, the distributions of SG3 and CP2 on the PVM are complementary with limited overlap (Fig 5H). Consistent with this finding, IEM results revealed SG3 expression in the middle to upper region of the PVM in both trophozoites and meronts (Fig 5M). In contrast, DG7-HA expression was mainly punctate in both trophozoites and meronts (Fig 5I). Additionally, low-level DG7 expression was detected in the PVM, partially colocalizing with CP2 in 3D images (Fig 5J). This suggests that DG7 also translocates to the PVM. The IFA and IEM results also showed colocalization of DG8 and CP2 in the PVM. In IFA, DG8 exhibited a ring-like staining around trophozoites and meronts, as well as additional expression near the parasite nuclei. There was partial colocalization with VVL and nearly complete colocalization with CP2 in the PVM (Fig 5K and 5L). IEM confirmed the basal localization of DG8 in the PVM (Fig 5N) and revealed additional DG8 expression on the nuclear membrane of merozoites (S6D Fig). To determine the relative positions of these proteins on the PVM, we measured the distance from the FO to the bottom of their expression. The results showed that the distance of DG8 expression on the PVM (501.0 ± 30.5 nm) was significantly shorter than that of CP2 expression (1,119.0 ± 65.1 nm) (S6E Fig). Additionally, SG3 expression (1,801.0 ± 100.2 nm) was located far from the FO (S6F Fig). Taken together, these data suggest that the SG3, DG7, and DG8 are secreted onto different areas of the PVM during parasite development.

### SG3 is also localized to two distinct structures outside the PVM and is not essential to them

**Fig 6:**
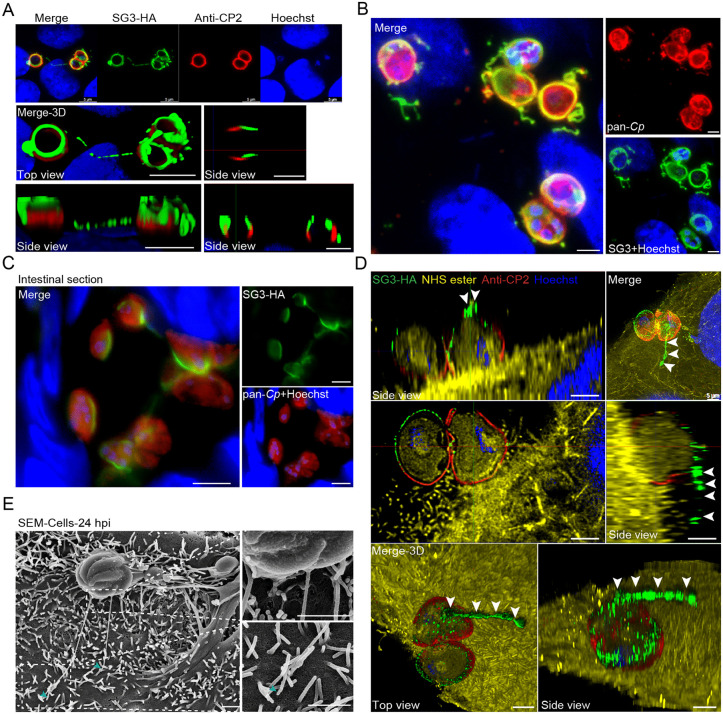
SG3-HA expression in a filament-like structure outside of the parasitophorous vacuole. **(A)** Immunofluorescence localization of SG3-HA expression in *Cryptosporidium parvum* grown in HCT-8 cells at 24 h post-infection (hpi) after staining with rabbit anti-HA (green), anti-CP2 (red), and Hoechst (blue). The same three parasites are shown at different magnifications and views. Scale bars, 5 μm. **(B)** Immunofluorescence analysis of SG3-HA stages in HCT-8 cells at 36 hpi, after staining with rat anti-HA (green), anti-*Cp* (red), and Hoechst (blue). Scale bars, 2 μm. **(C)** Immunofluorescence analysis of SG3-HA in the ileum of GKO mice infected with SG3-HA, after staining with anti-HA (green), anti-*Cp* (red), and Hoechst (blue). Scale bars, 5 μm. **(D)** Ultrastructure expansion microscopy (U-ExM) of the SG3-HA expression co-stained with anti-CP2 antibody in two trophozoites. SG3-HA infected cells (24 hpi) were fixed, expended in water-based gel, and stained with rat anti-HA antibody (green), anti-CP2 antibody (red), NHS ester that recognizes all proteins (yellow), and Hoechst (blue). The yellow hair-like structures on the surface of the host cell stained by NHS ester are microvilli. Scale bars, 5 μm. **(E)** Scanning electron microscopy of the HCT-8 cells infected with *C. parvum* for 24 hours. The filament-like structures are indicated by arrowheads. Scale bars, 1 μm.

Studies of SG3 localization revealed two unique structures that were labeled by SG3. One of them was a SG3-positive filamentous structure adjacent to the parasite (Fig 6A). Three-dimensional reconstructions of the Z-stack images showed that this structure was in close proximity to the host cells (Figs 6A and S7A). Second, the SG3-HA signal was found to be enriched on the surface of the previously described knob-like protrusions on the PVM. Both the filamentous and the knob-like SG3-positive structures were stained by the pan-*Cp* antibodies, but not by the VVL or anti-CP2 antibodies (Figs 6A and 6B, S7A and S7D). In HCT-8 cultures, SG3-positive knob-like and filamentous structures appeared at 1 and 2 h post-infection (hpi), respectively (S6C Fig).

To examine SG3 expression *in vivo*, we performed IFA and IEM of SG3-HA-infected mouse ileum. SG3 accumulated primarily at the top of the PVM with filament-like extensions, with the highest signal at the base of these extensions (Fig 6C). In IEM analysis, the gold particle-positive filaments resembled a modified microvillus-like structure (S7I Fig). We also performed SEM analysis of HCT-8 and enteroid cultures infected with the WT strain. We observed knob-like structures on the surface of the developmental stages, as well as the filament-like structures extending from the PVM to the microvilli of host cells or to the PVM of neighboring parasites (Figs 6E and S7B, S7C). To determine if these filament-like structures were modified microvilli, we performed IFA on SG3-HA-infected HCT-8 cultures using anti-HA and anti-ezrin antibodies as well as F-actin-reactive phalloidin. The anti-ezrin antibody and phalloidin stained the microvilli but neither reacted with the SG3-positive filaments (S7E and S7F Fig). Additionally, we used U-ExM to examine the filament-like structure closely. The results indicated that the SG3-positive filaments derived from the upper part of the parasite (Figs 6D, and S7G and S7H).

To determine whether the knob- and filament-like structures depend on *SG3*, we knocked out the *SG3* gene and replaced it with an mNeonGreen expression cassette (S8A Fig). We then assessed the phenotype using pan-*Cp* staining. Successful gene deletion was confirmed by diagnostic PCR (S8B Fig), and all oocysts from the *ΔSG3* line exhibited green fluorescence (S8C Fig). Using the SG3-HA line as a positive control, pan-*Cp* staining revealed that intracellular parasites from both the SG3-HA and *ΔSG3* lines possessed knob- and filament-like structures (S8D and S8E Fig). Additionally, we assessed the impact of the *SG3* deletion on *C. parvum* infection. Although deleting the *SG3* gene did not significantly impair parasite proliferation *in vivo* (S8F Fig), but it resulted in slightly improved body weight and survival of infected mice (S8G and S8H Fig).

## Discussion

Apicomplexan parasites, including *Cryptosporidium*, form a PVM to establish infection, yet the underlying molecular mechanisms are poorly understood. Using reverse genetics, we characterized the localization, trafficking, and function of CP2, a putative PVM protein in *Cryptosporidium*. We discovered that endogenously tagged CP2 is stored in the SG within sporozoites and merozoites, is secreted to the parasite-host interface during invasion, and is subsequently translocated to the PVM. However, deleting CP2 had a negligible effect on parasite growth *in vitro* and *in vivo*, and only slightly reduced the pathogenicity of a highly virulent *C. parvum* strain. Transcriptional profiling revealed the upregulation of other PVM-associated proteins, such as SG3, DG7 and DG8, in CP2-knockout parasites. Interestingly, SG3 is another SG protein and also localizes to two PVM modifications: a knob-like structure on the PVM and filament-like structure extending from the parasite (S9 Fig). Together, these findings suggest that the secretion of SG protein to the parasite-host interface during invasion. Their subsequent localization to the PVM suggests that they are probably involved in establishing an intracellular niche for *Cryptosporidium*. Nevertheless, direct validation through future gain- or loss-of-function experiments is required to confirm this hypothesis.

In this study, we have identified CP2 as a highly expressed SG protein. The SG is a newly discovered, *Cryptosporidium*-specific organelle whose size and expression profile differ from those of the canonical DG [10]. Although a spatial proteomic analysis identified 23 SG proteins, only the localization of SG1 and SG2 has been validated experimentally. However, CP2 was not among the 23 SG proteins identified. Two recent studies using endogenous gene tagging identified two other SG proteins: MVP1, encoded by cgd6_40, and SKSR1 encoded by cgd1_140 [19,23]. Therefore, CP2 is the fifth confirmed SG protein. Among these proteins, MVP1, CP2, and SG3 possess mucin-like features with threonine and serine tracts. Several of the SG proteins, including SG1, MVP1, and CP2, are highly expressed during the sporozoite and intracellular stages. Others, such as SG2, SKSR1, and SG3, are expressed at lower levels during these stages.

CP2 is secreted to the parasite-host interface during host cell invasion. Although it is stored in the SG, CP2 translocates from the middle region of the sporozoite to the apical end upon host cell attachment. There, it is secreted to the parasite-host interface and forms a ring-like pattern. Similar translocation and secretion processes have been observed in SG1, MVP1, and SKSR1. These findings suggest that these early secreted SG proteins may be necessary for host cell invasion and PV establishment [10,19,23]. However, CP2 and other SG proteins are also continuously secreted during intracellular development [10]. The rapid translocation of CP2 to the nascent PVM indicates that it may contribute to the early events of PV biogenesis, as observed in *T. gondii* [24]. However, its non-essential nature indicates either functional redundancy or a subtle role, both of which warrant further investigation. Despite the clear identification of CP2 in the SG of sporozoites and merozoites, CP2 has not been detected in any secretory organelles within trophozoites, microgamonts, or macrogametes. Therefore, different mechanisms are probably involved in the translocation and secretion of SG proteins in different developmental stages. The continuous secretion of SG proteins by intracellular stages, however, may involve the same secretory pathway for the constitutional secretion of DG in *T. gondii* [25]. Therefore, removing the signal peptide resulted in CP2 accumulation in the cytosol of intracellular stages but did not appear to affect its localization in sporozoites.

This study confirms that CP2 is a PVM protein but undergoes through dynamic translocation during the intracellular development of *Cryptosporidium*. The PVM is an integral part of the PV and plays a crucial role in parasite survival and replication, yet few PVM proteins of *Cryptosporidium* have been explicitly characterized. Several PV proteins have been identified, including SG2, ROP4, ROP7 and DG4 [7,10]. However, their precise localizations remain poorly understood. Using IFA and IEM analyses of endogenously tagged parasites, we provide clear evidence indicating that once secreted into the interface, CP2 localizes to the entire surface of the nascent PV in young trophozoites. In later stages, particularly in mature meronts and macrogametes, however, CP2 localizes to the lower part of the PVM. This localization corresponds with the radial folds at the base of the PVM, forming a distinctive “cupcake-like” structure at the parasite-host cell interface. These folds are often supported by elongated host microvilli [9,26]. The near-interface localization of CP2 suggests that this protein may mediate parasite-host interactions. CP2’s multiple localizations suggest that the composition of PVM undergoes dynamic changes as the nascent PVM matures. This is likely due to the regulated secretion of SG and DG proteins during invasion and the constitutional secretion of SG proteins during intracellular development, as well as the parasite’s adaptive responses to the host environment. Consistent with this observation, a previous study showed CP2 expression in multiple membrane structures of intracellular stages of *C. parvum* [15]. These dynamic changes in PVM-associated proteins are not observed in related apicomplexans such as *Plasmodium* and *Toxoplasma* [27,28]. However, *Cryptosporidium* utilizes the attachment zone as the primary interface between the parasite and the host cell and relies on both SG and DG proteins as the primary components of the PVM.

Interestingly, despite being an integral component of the PVM, depleting CP2 does not appear to significantly impact the growth of a highly virulent *C. parvum* strain. Parasites lacking *CP2* exhibit normal proliferation and morphology, suggesting that CP2 does not play a direct role in invasion and growth. This finding is consistent with observations in *T. gondii*, where most of PVM proteins are dispensable [29]. The upregulation of three subtelomeric genes—specifically, two genes (SG3 and DG7) adjacent to the CP2 locus and another on chromosome 8—could be an adaptive response or an artifact of genetic manipulation, and its functional relevance merits further study. Notably, both SG3 and CP2 are secretory proteins stored in SG prior to secretion to the parasite-host interface. In contrast, DG7 and DG8 are DG proteins. Together, the spatial localization of these three proteins (SG3, DG7, and DG8) spans nearly the entire PVM. Therefore, SG proteins, in addition to DG proteins, are significant components of *Cryptosporidium* PVM. The small *Cryptosporidium* genome harbors multiple paralogous genes that encode several families of secretory proteins, including SKSR, insulinase-like proteases (INS), mucins, and MEDLE proteins [30]. These genes are typically clustered in subtelomeric regions on multiple chromosomes. Consequently, they are highly polymorphic and exhibit copy number variation, hallmarks of rapid evolution driven by recombination and their roles in invasion, pathogenesis, and host adaptation [31–33]. Thus, the redundancy in PVM proteins is likely another strategy used by *Cryptosporidium* to safeguard its intracellular niche.

Although the deletion of CP2 led to increased expression of SG3 and DG8, the underlying mechanisms remain unknown. The PVM localization of DG8 largely overlaps with that of CP2, whereas SG3 exhibits a distinct, apical distribution. We propose that this distinct partitioning is not contradictory but rather reflects a sophisticated functional specialization within the PVM that is directly aligned with the unique extracytoplasmic, intracellular niche of *Cryptosporidium*. The PVM is likely not a uniform structure but is functionally polarized into distinct domains. Since *C. parvum* resides in an intracellular but extracytoplasmic compartment that is typically surrounded by host microvilli, we hypothesize that CP2 and DG8 (localized to the basal PVM) and SG3 (localized to the apical PVM) may interact with the host cell differently. Additionally, the genetic complementarity between non-essential genes may involve a unified response that operates across different intracellular organelles.

In addition to the PVM, SG3 is further expressed in two unique parasitophorous vacuole extensions: a knob-like structure and a filament-like structure. we initially identified these structures via IFA analysis of SG3-HA using an anti-HA antibody, and then confirmed their existence using pan-*Cp* polyclonal antibodies. Additionally, SEM has further revealed analogous surface structures outside the PVM. The filament-like structure near the parasites suggests potential roles in intercellular communication or material exchange. However, the origin and nature of the SG3-positive filament-like structures remain to be fully determined. They may represent parasite-derived extensions; however, we cannot rule out alternative possibilities, such as trails of shed SG3 protein. Future studies, such as live-cell imaging, will be necessary to distinguish between these models. The filament-like structure of *C. parvum* is resemble the PVM projections (PVMPs) in *T. gondii*, in which DG proteins (GRA3, GRA7, and GRA14) localize to form filamentous extensions of the PVM into the host cytoplasm. PVMPs are hypothesized to mediate vesicle or organelle docking to the PVM [34,35]. In *Plasmodium*, an analogous structure called the tubulovesicular network (TVN) facilitates nutrient uptake [36,37]. Notably, TVN-like structures have been observed in hypnozoites, occasionally associating with host nuclei [38]. Deleting the SG3 gene apparently does not affect the formation of the knob-like and filamentous structures. Further investigation is therefore warranted to understand SG3 secretion into these structures and its potential role in host-pathogen interactions. Answers to these questions will improve our understanding of the intracellular adaptation of *Cryptosporidium* and other apicomplexans.

## Materials and methods

### Animal studies and ethic approval

All mouse studies were approved by the Animal Use Committee of the South China Agricultural University under the protocol No. 2022c070. Interferon-γ-knockout (GKO) mice with a C57BL/6 background were purchased from the Institute of Laboratory Animals Science, Chinese Academy of Medical Sciences and bred in-house in a specific pathogen-free animal facility. The GKO mice were used in infection experiments at 3–5 weeks age. All studies followed the practices recommended in the Guide for Care and Use of Laboratory Animals [39].

### *Cryptosporidium* strain

The *Cryptosporidium parvum* IIdA20G1-HLJ isolate was originally obtained from a dairy calf during a cryptosporidiosis outbreak in China [40] and maintained and propagated in the laboratory by sequential passages in GKO mice as previously described [41]. Oocysts were purified from the feces using discontinuous sucrose and cesium chloride gradients [42] and stored at 4°C in phosphate-buffered saline (PBS) containing 1 mg/mL ampicillin, 1 mg/mL streptomycin, and 0.5 mg/mL vancomycin for up to three months after the fecal collection. The subtype identity of this isolate was confirmed after each passage by sequence analysis of the 60 kDa glycoprotein (GP60) genes [43]. Prior to infection, the oocysts were treated with 1.25% sodium hypochlorite on ice, washed three times with PBS containing 1% BSA, and resuspended in 1% BSA-PBS. To obtain free sporozoites, bleached oocysts were excysted in 0.75% sodium taurocholate in PBS at 37°C for 1 h.

### Cell culture

The human ileocecal adenocarcinoma cell line HCT-8 (ATCC, CCL-244) was obtained from the Shanghai Branch of the Chinese Academy of Sciences. The cells were cultured in RPMI 1640 medium (Gibco, Grand Island, NY, USA) supplemented with 10% fetal bovine serum (ExCell Bio, Suzhou, China) and 1% penicillin-streptomycin solution (Gibco) at 37°C in 5% CO_2_.

### Bioinformatic analyses

The amino acid sequences of the cgd6_5410, cgd6_5400, cgd6_5420, and cgd8_5300 genes were extracted from the genome of IIdA20G1-HLJ (JBJGDY000000000). The structure of these proteins and functional domains were predicted using SMART (http://smart.embl.de/). Signal peptide prediction was performed using the SignalP 5.0 server (https://services.healthtech.dtu.dk/services/SignalP-5.0/) [44]. Motif prediction was performed using MEM-Suite (https://meme-suite.org/meme/tools/meme) and PROSITE (https://prosite.expasy.org/) [45]. Consensus prediction of membrane protein topology was performed using ProtScale (https://web.expasy.org/protscale/) [46]. Disordered regions in the sequences were predicted using PONDR (http://www.pondr.com/) [47]. O-linked glycosylation sites were predicted by NetOGlyc - 4.0 (NetOGlyc 4.0 - DTU Health Tech - Bioinformatic Services) [48].

### Construction of CRISPR/Cas9 plasmids for gene tagging and deletion

Transgenic parasite lines were generated as described [49,50]. The Cas9 plasmid was generated by adding the guide RNA targeting the *CP2*, *SG3*, *DG7* or *DG8* designed using the eukaryotic pathogen CRISPR guide RNA/DNA design tool (http://grna.ctegd.uga.edu) into a linear Cas9 backbone amplified from pACT:Cas9-GFP, U6:sgINS1 [51] via Gibson assembly using the ClonExpress II one-step cloning kit (Vazyme, Nanjing, China). To knock out the *CP2* gene or its signal peptide, the Cas9 plasmid was generated by adding two gRNAs targeting the N-terminus and 3’ UTR of *CP2* into the linear Cas9 backbone. The Cas9 plasmid used to knock out the *SG3* gene was same as the one used in tagging *SG3 gene*.

To construct homology repair plasmids for tagging genes, approximately 400–1,000 bp segments of the C-terminus and 3’ UTR of target genes were amplified from *C. parvum* genomic DNA, while fragments of the pU19 backbone and 3HA-Nluc-Neo were amplified from the pINS1–3HA-Nluc-neo plasmid [51]. These four fragments were assembled to form the tagging plasmid using Gibson assembly. To construct the gene knockout plasmid, the 5’ UTR and 3’ UTR sequences of *CP2* or *SG3* were amplified from *C. parvum* genomic DNA and pU19 backbone and Nluc-Neo fragments were amplified from the pINS1–3HA-Nluc-neo plasmid. These four fragments were assembled using Gibson assembly. To construct the plasmid for deleting the CP2 signal peptide, the nucleotide sequence of CP2 without the signal peptide was amplified from *C. parvum* genomic DNA, and assembled together with the 3HA-Nluc-Neo, the 5’ UTR and 3’ UTR sequences of *CP2* fragments, and the pU19 backbone. All primers were synthesized by Sangon Biotech (Shanghai, China). They and the plasmids used or constructed in this study are listed in S1 Table.

### Generation of transgenic parasites

Sporozoites were electroporated with the Cas9 plasmid and the gene tagging or deletion plasmid. Briefly, 8 × 10^7^ sporozoites were suspended in SF buffer (Lonza, Basel, Switzerland) and co-electroporated with 50 μg tagging or deletion plasmid and 50 μg Cas9 plasmid using the AMAXA 4D nucleofector system (Lonza) with the program of EH100. Immediately thereafter, the electroporated sporozoites were gavaged into a GKO mouse that had been orally gavaged with 200 μL of 8% (wt/vol) sodium bicarbonate 5 min prior to infection. To select for transgenic parasites, the mouse was treated with 16 g/L paromomycin (Yuanye, Shanghai, China) in drinking water starting at 16 h post infection (hpi) throughout the infection. For the amplification of transgenic lines, GKO mice were gavaged with 1 × 10^3^ transgenic oocysts and treated with paromomycin in drinking water immediately after infection. Fecal pellets were collected from infected mice starting at 6 days post-infection (dpi) and stored at 4°C.

### *In vitro* and *in vivo* infection studies

To measure the parasite growth *in vitro*, HCT-8 cells were seeded in 24-well plates and cultured as described above, followed by infection with 1 × 10^5^ bleached oocysts/well. To monitor the parasite burden in HCT-8 cells, cells were harvested at 2, 12, 24, 36 and 48 hpi for luciferase measurement. To measure the parasite shedding *in vivo*, 3- to 5-week-old GKO mice were randomly divided into groups: CP2-HA, *ΔCP2,* uninfected, SG3-HA and *ΔSG3*. The mice were housed in individual cages (one mouse per cage) throughout the course of the infection. Each mouse was gavaged with 1 × 10^3^ transgenic oocysts and received paromomycin (16 g/L) in drinking water immediately after infection. Fecal samples were collected at two-day intervals and stored at 4°C for the luciferase assay. The mice were weighed every two days, and the time of death was recorded to construct a survival curve.

### Luciferase assay

Luciferase activity was measured using the Nano-Glo Luciferase Assay (Promega, Madison, WI, USA). Fecal pellets were transferred to 1.5-mL microcentrifuge tubes containing ten 3-mm glass beads (Themo Fisher Scientific, Waltham, MA, USA) and 1 mL of fecal lysis buffer (50 mM Tris, pH 7.6; 2 mM DTT; 2 mM EDTA, pH 8.0; 10% glycerol; and 1% Triton X-100) [52]. The samples were homogenized using a FastPrep nucleic acid extractor (MP Biomedicals, Santa Ana, CA, USA) at 6.0 m/s for 45 sec and then centrifuged at 19,000 × *g* for 1 min. Fifty microliters of the supernatant was added to one well of a 96-well plate (Themo Fisher Scientific), mixed with 50 μL of Nano-Glo luciferase buffer containing substrate, and incubated at room temperature (RT) for 3 min. Luminescence levels were read using a Synergy HT multimode reader (BioTek, Winooski, VT, USA). For luciferase analysis of cell cultures, 100 μL of Nano-Glo luciferase buffer was added to the wells of 24-well plates. After incubating at 37°C for 10 min, the suspension was transferred to a 96-well plate. Then, 1 μL of Nano-Glo luciferase substrate was added, and the plate was incubated at RT for 3 min. Finally, luminescence levels were read on a Synergy HT multimode reader (BioTek).

### Confirmation of gene modifications by PCR

Genomic DNA was extracted from 100 mg of feces from transgenic parasite-infected mice using the FastDNA SPIN Kit for Soil (MP Biomedicals) and stored at -80°C. PCR analysis was used to confirm the genomic insertion, employing the purified DNA as the template, Phanta Max Super-Fidelity DNA Polymerase mix (Vazyme), and primers listed in S1 Table. The PCR products were examined by agarose gel electrophoresis and viewed using the UVP ChemStudio 815 Bundle PROMO imaging system (Analytik Jena, Germany).

### Production of polyclonal antibodies against CP2 and *C. parvum*

To express recombinant CP2 (rCP2) protein in *Escherichia coli*, the CP2 coding sequence (cgd6_5410) was amplified by PCR using genomic DNA from *C. parvum* and the primers listed in S1 Table. The PCR product (2073 bp) was cloned into the pCold I expression vector (Fenghui, Hunan, China) to express rCP2 with 6× His tags at the N-terminus, and the accuracy of the positive clone was confirmed by DNA sequencing. The pCold I-CP2 plasmid encoding a 6× His-tagged CP2 was transformed into *E. coli* BL21(DE3) pLysS cells (WEIDI, Shanghai, China). The transformed *E. coli* was grown in LB medium containing 50 μg/mL ampicillin sulfate until the optical density at 600 nm (OD_600_) reached 0.6-0.8. The recombinant protein expression was induced with 1 mM IPTG at 15°C for 16 h. To purify rCP2, the cells were sonicated, and the supernatant was removed by centrifugation at 15,000 × *g*. The pellet containing rCP2 was suspended in 8 M urea-Tris buffer and incubated overnight at 4°C. The rCP2 protein in the suspension was then purified using Ni-nitrilotriacetic acid (Ni-NTA) Superflow resin (GE Healthcare/Cytiva, Marlborough, MA, USA) and eluted with 200 mM imidazole-Tris buffer. The purity of the eluted fractions was assessed by 10% SDS-PAGE, and the identity of rCP2 was confirmed by Western blotting using the anti-His antibody (Santa Cruz Biotechnology, USA).

To generate polyclonal antibodies against CP2, two New Zealand White rabbits were immunized subcutaneously with 200 μg of rCP2 in an equal volume of Freund’s complete adjuvant (Sigma-Aldrich, St. Louis, MO, USA) for the primary immunization, followed by two boosters of 100 μg of rCP2 in Freund’s incomplete adjuvant (Sigma-Aldrich) at 10- to 14-day intervals. Sera were collected 10 days after the last immunization, and the polyclonal antibodies (pAb) to CP2 were affinity-purified using Protein A (Sigma-Aldrich).

To generate pAbs against *C. parvum* (*Cp*), 4 × 10^7^ excysted sporozoites were lysed by freeze-thaw treatment 6 times (3 min in liquid nitrogen, and 3 min at 37°C). One rabbit was immunized subcutaneously with 500 μg of the crude antigen in Freund’s complete adjuvant (Sigma-Aldrich), followed by three boosters at 21-day intervals with 200 μg of antigen in Freund’s incomplete adjuvant (Sigma-Aldrich). Sera containing the pan-*Cp* antibodies were harvested from the rabbit and used in immunofluorescence assays at a dilution of 1:2,000.

### Western blot analysis

HCT-8 cells were grown to confluence in 6-well plates and infected with 5 × 10^6^ oocysts/per well transgenic or wild-type oocysts for 48 h. Cells were lysed in RIPA buffer (Beyotime) for 5 min and centrifuged at 13,000 × *g* for 10 min. The supernatant together with 5 × SDS-PAGE sample loading buffer (YEASEN, Shanghai, China) was boiled for 5 min, separated by SDS-PAGE, and transferred onto the nitrocellulose membrane. The membrane was blocked with 5% nonfat milk in PBS and probed with rabbit anti-HA antibodies (Cell Signaling Technology, 3724S) diluted 1:1,000 in PBST, or anti-EF1α [53] diluted 1:100 in PBST, followed by secondary antibodies conjugated with HRP reagents (Beyotime) diluted in 1:1,000 in PBST. The blots were visualized and imaged using the UVP ChemStudio 815 Bundle PROMO imaging system (Analytik Jena).

### Immunofluorescence microscopy (IFA)

HCT-8 cells were plated on 12-mm glass coverslips in 24-well plates and cultured to confluence. They were infected with 4 × 10^5^ sporozoites per well for 15 min, 30 min, 1 h, 2 h, 24 h or 48 h. The cultures and sporozoites were fixed in 4% formaldehyde-PBS for 15 min at RT. After three washes with PBS, the fixed cells were permeabilized in PBS containing 0.5% Triton X-100 at RT for 15 min and blocked with 5% BSA-PBS at RT for 30 min. They were treated overnight at 4°C with primary antibodies diluted with 1% BSA-PBS, including rat anti-HA (Roche, Basel, Switzerland) at 1:800, rabbit anti-HA (Cell Signaling Technology, 3724S) at 1:1,000, rabbit anti-EF1α at 1:200, rabbit anti-CP2 at 1:2,000, anti-ezrin at 1:200 (Cell Signaling Technology, 3145S), phalloidin at 1:1000 (Yeasen, Shanghai, China, 40762ES75), or rabbit pan-*Cp* at 1:2,000. After three washes with PBS, the cells were incubated at 37°C for 60 min with Alexa Fluor-conjugated secondary antibodies (Thermo Fisher Scientific) diluted 1:500 in 1% BSA-PBS, fluorescein-conjugated *Vicia villosa* lectin (VVL) (Vector; Newark, NJ, USA) diluted 1:500, or fluorescein-conjugated Sporo-Glo polyclonal rat IgG antibody diluted 1:20 (Waterborne, Inc., New Orleans, LA, USA). Nuclear DNA was stained with Hoechst (Thermo Fisher Scientific) diluted 1:1,000 in PBS for 20 min. Finally, the coverslips were washed three times with PBS and once with water, and examined by florescence microscopy. For analysis of the parasites in tissues, the small intestine of infected mice was excised and “Swiss rolled” prior to overnight fixation in formalin. Sections were prepared by Servicebio (Wuhan, China) using routine procedures, and stained in-house with rat anti-HA diluted 1:400 and rabbit pan-*Cp* diluted 1:1,000 in 1% BSA-PBS, following by Alexa Fluor-conjugated secondary antibodies and Hoechst as described above. Imaging was performed using a BX53 microscope (Olympus, Tokyo, Japan) and a Stellaris 5 confocal microscope (Leica, Wetzlar, Germany). Images were acquired using cellSens and LAS X software.

### Ultrastructure expansion microscopy (U-ExM)

The U-ExM of transgenic sporozoites was performed following procedures previously described [19,54,55]. In brief, the sporozoites were transferred to poly-D-lysine-coated coverslips (12 mm) in 24-well plates, incubated at RT for 20 min, and fixed with 4% formaldehyde for 5 min. The sporozoites on the coverslips were further treated with 1 mL of an acrylamide-formaldehyde solution (2% acrylamide and 1.4% formaldehyde in PBS) at 37°C overnight to prevent cross-linking. The samples were then embedded in 20 μL of a water-based gel (19% sodium acrylate, 1% acrylamide, 0.1% N, N’-methylenebisacrylamide, 0.5% tetramethylethylenediamine, and 0.5% ammonium persulfate in PBS, all from Sigma) at 4°C for 5 min, incubated at 37°C for 1 h, and denatured at 95°C for 1 h. The samples were expanded in water to approximately four times the original size of the sporozoites. The gels were shrunk in PBS overnight to reduce the amount of antibodies and dye required for labeling. The gels were then incubated overnight at 4^o^C with a rat anti-HA (Roche, 11867423001) diluted 1:200 in 2% BSA, or a rabbit anti-CP2 diluted 1:150 in 2% BSA. After three washes with PBS, the samples were incubated for 3 h with Alexa Fluor 488-conjugated goat-anti-rat IgG (Thermo Fisher Scientific) diluted 1:200 and Hoechst (Thermo Fisher Scientific) diluted 1:250, both in PBS. The gels were then incubated in 10 mg/mL of Atto 565 NHS ester (Sigma-Aldrich) at 37^o^C for 3.5 h. Finally, the gels were re-expanded in water and examined under a Stellaris 5 confocal microscope (Leica). Images were acquired using LAS X software with a super-resolution lightning system.

### Immunoelectron microscopy (IEM)

GKO mice were gavaged with 1 × 10^4^ oocysts of transgenic CP2-HA, SG3-HA, DG7-HA, or DG8-HA lines. Tissue samples of the ileum were collected at 14 dpi, prefixed in 0.1% glutaraldehyde for 4 h at 4°C, followed fixation in 2% paraformaldehyde overnight at 4°C. The samples were washed four times in 0.1 M glycine, dehydrated in ethanol, and embedded in LR White resin (Sigma) at -20°C for 48 h. The imbedded samples were sectioned at 70 nm with a Leica ultramicrotome (EM UC7) and placed on single-mesh formvar-carbon-coated nickel grids. The sections on the grids were incubated with rabbit anti-HA antibody (Cell Signaling Technology) at 1:20 dilution at RT for 1 h and at 4°C overnight, followed by goat-anti-rabbit IgG conjugated with 10/12 nm colloidal gold (Sigma-Aldrich) at 1:20 dilution and 37°C for 1 h. The sections were stained with 2% uranyl acetate (SPI Supplies; West Chester, PA, USA) and 10% lead citrate (SPI Supplies) and examined under a Talos L120C electron microscope (Thermo Fisher Scientific).

### Scanning electron microscopy (SEM)

Air-liquid interface (ALI) culture of murine enteroids was established as previously described [56]. The cells in the transwells were infected with wild-type (WT) oocysts and cultured for 72 h. In addition, HCT-8 cells were infected with WT oocysts and cultured for 24 h. After washing with cold PBS, the cultures were fixed in the dark at 4°C with 2.5% glutaraldehyde for 48 h. The samples were fixed overnight at 4°C in 2.5% glutaraldehyde. After three rinses with PBS, the samples were fixed in 1% osmic acid for 2 h, rinsed three times with PBS, and dehydrated in 30%, 50%, 60%, 70%, 80%, 90%, and 100% ethanol for 15 min each. After drying in a Leica CPD300 desiccator, the samples were sprayed with EM ACE600 High Vacuum Sputter Coating (Leica) and examined under a field-emission scanning electron microscope (Verios 460, FEI, USA).

### RNA sequencing and bioinformatics analysis

Total RNA was extracted from HCT-8 cells infected with 1 × 10^5^ oocysts per well (48 well plates) the WT or *ΔCP2* line at 48 hpi using TRIzol (Thermo Fisher Scientific), and sent to Guangzhou Genedenovo Biotechnology Co. (Guangdong, China) for RNA sequencing (RNA-seq). RNA libraries were prepared using an Illumina TruSeq RNA Sample Prep Kit (Illumina) and sequenced using the 150 bp paired-end technique on an Illumina Novaseq 6000 (Illumina). Raw sequence reads were trimmed for adapter and low-quality sequences using Trimmomatic v0.39 and mapped to the reference genomes of *C. parvum* IOWAII using STAR v2.7.9a with default parameters. Expression matrices were generated from the RNA-seq data using RSEM version 1.3.1. Differentially expressed genes (DEGs) between the WT and *ΔCP2* lines were identified using the “DESeq2” package in R v4.1.0 with the following criteria: absolute fold change > 1.5 and *P* values < 0.05. Differences in gene expression were validated by quantitative reverse transcription-PCR (qRT-PCR) analysis of total RNA extracted from HCT-8 cell cultures infected with the WT and *ΔCP2* lines for 48 h. For this, cDNA was synthesized from the RNA using Hifair Ⅲ 1st Strand cDNA Synthesis SuperMix (Vazyme) and subjected to qPCR of target genes using the Hieff qPCR SYBR Green Master Mix (Vazyme) and primers listed in S1 Table. *SSU* rRNA expression was used as an internal control and each analysis was performed in triplicate. The qPCR was performed on a LightCycler 480 Instrument II (Roche) using the following conditions: pre-denaturation at 95°C for 3 min; denaturation at 95°C for 5 s, annealing at 55°C for 10 s, and extension at 72°C for 40 s for a total of 50 cycles; melting curve analysis at 95°C for 10 s, 50°C for 30 s, and 90°C for continuous; cooling at 40°C for 30 s.

### Statistical analysis

A two-way ANOVA test followed by Sidak’s multiple comparisons was used to evaluate differences between the experimental and the control group (defined in each figure legend). For the qRT-PCR data, the gene expression was compared between the *ΔCP2* and WT groups using the two-tailed Mann-Whitney U test. Differences were considered significant at *P* ≤ 0.05. The statistical analyses were performed using GraphPad Prism v10, and details on the number of biological replicates and specific statistical tests are provided in the figure legends.

## Supporting information

S1 Fig Sequence features of CP2 of *Cryptosporidium parvum.* **(A)** Schematic representation of disordered regions within the CP2 protein sequence as predicted by PONDR. Values > 0.5 correspond to disordered regions, while values < 0.5 correspond to folded regions. **(B)** Distribution of putative O-linked glycosylation sites (red residues) predicted by NetOGlyc 4.0. **(C)** Schematic representation of the hydrophobicity within CP2 predicted by Expasy-ProtScale. The horizontal axis represents the amino acid position of the protein, and the vertical axis represents hydrophobicity, with larger values indicating greater hydrophobicity.
(TIF)

S2 Fig Ultrastructural localization of CP2-HA in merozoites. Meronts in the ileal tissue from mice infected with CP2-HA were examined using immunoelectron microscopy with rabbit anti-HA, followed by 10-nm colloidal gold-conjugated goat-anti-rabbit IgG. Gold particles were primarily observed in small granules within merozoites and in the lower part of the parasitophorous membrane (PVM). N, nucleus; M, microneme; SG, small granule; DG, dense granule; R, rhoptry. Scale bars, 500 nm.
(TIF)

S3 Fig CP2 (cgd6_5410) localization in the parasitophorous vacuole membrane of intracellular stages of *C. parvum.* **(A)** Immunofluorescence localization of CP2-HA in intracellular stages. HCT-8 cells were infected with CP2-HA parasites for 48 h post-infection (hpi), and stained with rabbit anti-HA (red), *Vicia villosa* lectin (VVL, green), and Hoechst (blue). The microgamont is indicated by a white arrowhead. Scale bars, 2 μm. **(B-D)** Ultrastructural localization of CP2-HA in the trophozoite (B), microgamont (C), and macrogamete **(D)** within the mouse ileum, using immunoelectron microscopy with rabbit anti-HA, followed by 10-nm colloidal gold-conjugated goat-anti-rabbit IgG. Black arrowheads indicate the distribution of gold particles. PM, parasites membrane; PVM, parasitophorous vacuole membrane; HM, host membrane; N, nucleus. Scale bars, 200 nm.
(TIF)

S4 Fig Deletion of the signal peptide prevents the secretion of CP2. **(A)** Schematic for deleting the signal peptide of *CP2* using CRISPR/Cas9. The two single guide RNA (sgRNA) targeting the N-terminus and 3’UTR of the *CP2* gene, repair template for homologous recombination, and primers used for diagnostic PCR are shown. Nluc, nanoluciferase; neo, neomycin resistant marker; *pEno*, enolase promoter. **(B)** PCR confirmation of the correct integration of the repair template in the *CP2ΔSP*-HA line. Genomic DNA from wild-type (WT) and *CP2ΔSP*-HA transgenic parasites were used in PCR. The locations of primers used to verify the 5’ ins and 3’ ins are indicated in (A), and an irrelevant gene (cgd2_920) was used as the DNA control. **(C)** Immunofluorescence localization of *CP2ΔSP*-HA in the sporozoite after staining with rabbit anti-HA (green), anti-EF1α that recognizes whole parasite (red), and Hoechst (blue; scale bars, 2 μm. **(D)** Immunofluorescence localization of *CP2ΔSP*-HA in trophozoites (1 nucleus or 1N), meronts (2N, 4N and 8N (two white dotted circles), marcogamete, and microgamont (white dotted circle) grown in HCT-8 cells. The cells were fixed at 48 hours post-infection (hpi) and stained with rabbit anti-HA (red), *Vicia villosa lectin* (VVL, green), and Hoechst (blue). Scale bars, 2 μm.
(TIF)

S5 Fig Localization of SG3, DG7 and DG8 expression in *C. parvum.* **(A-C)** Schematic of the endogenous tagging of the cgd6_5400 (*SG3*), cgd6_5420 (*DG7*) and cgd8_5300 (*DG8*) genes with the 3HA tag using CRISPR/Cas9. A single guide RNA (sgRNA) targeting the C-terminus of the target gene, repair template for homologous integration, and primers used in diagnostic PCR are shown. Nluc, nanoluciferase; neo, neomycin resistant marker; *pEno*, enolase promoter. **(D-F)** Confirmation of correct integration of the repair templates in the SG3-HA, DG7-HA, and DG8-HA lines by PCR. Genomic DNA from wild type (WT) and transgenic parasites were used in PCR. The locations of the primers used to verify the 5’ (5’ ins) and 3’ (3’ ins) integrations are indicated in (A-C), and an irrelevant gene (cgd2_920) was used as the DNA control. **(G)** Immunofluorescence colocalization of CP2 and SG3-HA in the sporozoite after staining with rat anti-HA (green), anti-CP2 antibody (red), and Hoechst (blue); scale bars, 2 μm. **(H and I)** Ultrastructure expansion microscopy (U-ExM) of the SG3-HA expression co-stained with anti-CP2 antibody in sporozoites. SG3-HA sporozoites or SG3-HA infected cells (24 hpi) were fixed, expended in water-based gel, and stained with rat anti-HA (green), anti-CP2 antibody (red), NHS ester that recognizes granules (grey), and Hoechst (blue). Scale bars, 5 μm. **(J-L)** Ultrastructural localization of SG3-HA (J), DG7-HA (K), and DG8-HA (L) expression in merozoites using immunoelectron microscopy (immuno-EM) of the ileum of infected mice with rabbit anti-HA, followed by 12-nm colloidal gold-conjugated goat-anti-rabbit IgG. Scale bars, 200 nm. **(M)** Sizes of HA-positive vesicles in immuno-EM of SG3-HA, DG7-HA, and DG8-HA merozoites.
(TIF)

S6 Fig SG3-HA translocation during invasion and early growth of *C. parvum.* **(A and B)** Dynamics of SG3-HA and DG8-HA secretion during invasion and early growth of *C. parvum* as revealed by immunofluorescence microscopy. HCT-8 cells were infected with SG3-HA or DG8-HA sporozoites, fixed at 15 min, 30 min, 1 h and 2 h post-infection (hpi), and stained with rabbit anti-HA (green), anti-*Cp* that recognizes whole parasites (red), and Hoechst (blue). Scale bars, 2 μm. **(C)** Appearance of SG3-positive knob- or filament-like structures (indicated by white arrowheads) in trophozoites at in 1 hpi and 2 hpi. Scale bars, 2 μm. **(D)** DG8-HA expression in the lower PVM (highlighted blue) and the nuclear membrane (blue dotted circles) of an immature meront (2N) in the ileum under immuno-EM. N, nucleus. Scale bars, 200 nm. **(E)** The PVM length (mean ± SEM) labeled by CP2 and DG8. The *P* value was calculated using a two-tailed Mann-Whitney test. **(F)** The length (mean ± SEM) between the feeder organelle and the lower part of the SG3-positive PVM.
(TIF)

S7 Fig SG3-HA localization in the filament-like and knob-like structures outside the parasitophorous vacuole. **(A)** Immunofluorescence localization of SG3-HA in parasites grown on HCT-8 cells, showing top and side views. The cells were fixed at 24 hpi and stained with rat anti-HA (red), *Vicia villosa* lectin (VVL, green), and Hoechst (blue). Scale bars, 5 μm. **(B and C)** Scanning electron microscopy of the wild-type strain grown on HCT-8 cells for 24 h (B) and an enteroid-derived air-liquid interface (ALI) culture for 72 h (C). White arrowheads indicate knob-like structures on the surface of the parasitophorous vacuole and filament-like structures between parasites. Scale bars, 2 μm. **(D)** Immunofluorescence localization of SG3-HA in parasites grown on HCT-8 cells for 24 h using anti-HA (green), pan-*Cp* (red), and Hoechst (blue). White arrowheads indicate the knob-like structures. Scale bars, 2 μm. **(E)** Immunofluorescence localization of SG3-HA and microvilli in HCT-8 cultures infected for 24 h using anti-HA (green), anti-ezrin (red), and Hoechst (blue). Scale bars, 2 μm. **(F)** Immunofluorescence localization of SG3-HA and microvilli in parasites grown on HCT-8 cells for 24 h using anti-HA (green), phalloidin (red), and Hoechst (blue). Scale bars, 5 μm. **(G and H)** Ultrastructure expansion microscopy (U-ExM) of the SG3-HA expression co-stained with anti-CP2 antibody in sporozoites. SG3-HA infected cells (24 hpi) were fixed, expended in water-based gel, and stained with rat anti-HA (green), anti-CP2 antibody (red), NHS ester that recognizes all proteins (yellow), and Hoechst (blue). Scale bars, 5 μm. **(I)** Ultrastructural localization of SG3-HA in a trophozoite-like using immunoelectron microscopy with a rabbit anti-HA antibody and a 12-nm colloidal gold-conjugated goat-anti-rabbit IgG antibody. Scale bars, 200 nm.
(TIF)

S8 Fig *ΔSG3* is also dispensable in *Cryptosporidium parvum.* **(A)** Schematic for knocking out the *SG3* gene in *C. parvum*. The *SG3* gene was replaced by the Nluc-Neo cassette and mNeonGreen via the CRISPR/Cas9 method. The location of the one gRNA used is shown. Nluc, nanoluciferase; neo, neomycin resistance marker; *pEno*, enolase promoter. **(B)** Verification of the integration of the repair template in the *ΔSG3* line by PCR analysis of DNA extracted from wild-type (WT) and *ΔSG3* parasites. The locations of primers used to verify 5’ ins, 3’ ins, and CDS of the *SG3* gene are indicated in (A). **(C)** Oocysts of *ΔSG3* line. Scale bars, 5 μm. **(D and E)** Assessment of the effect of *SG3* deletion on the formation of the filament-like structure by IFA of HCT-8 cultures infected with SG3-HA or *ΔSG3* parasites for 24 h, using anti-HA antibody/mNeonGreen (green), pan-*Cp* that recognizes whole parasite (red), and Hoechst (blue) Scale bars, 5 μm. **(F)** Parasite burden of GKO mice infected with 1 × 10^3^ SG3-HA or *ΔSG3* oocysts, as indicated by luciferase activity in fecal pellets. Each bar represents the mean ± SEM of data from five GKO mice in one infection experiment. All mice in the infection experiment were housed separately from each other in individual cages (one mouse per cage). **(G and H)** Body weight changes (mean ± SEM, n = 5) and survival curve of GKO mice infected with SG3-HA or *ΔSG3* during the infection experiment, uninfected mice as control.
(TIF)

S9 Fig Localization of the secretory proteins identified in this study. **(A)** Illustration of CP2, DG8 and SG3 in the secretory organelles of the sporozoites and on the parasitophorous vacuole membrane of the intracellular stages. **(B)** Scanning electron microscopy images of the wild-type strain grown on HCT-8 cells for 24 h. White arrowheads indicate the filamentous structure on the surface of the parasitophorous vacuole and between parasites, supporting the placement of SG3-positive filamentous structure in the illustration. Scale bars, 2 μm.
(TIF)

S1 Table Primers, plasmids and transgenic parasites used in this study. (XLSX)

## References

[1] Checkley W, White AC Jr, Jaganath D, Arrowood MJ, Chalmers RM, Chen X-M, et al. A review of the global burden, novel diagnostics, therapeutics, and vaccine targets for cryptosporidium. Lancet Infect Dis. 2015;15(1):85–94. doi: 10.1016/S1473-3099(14)70772-8 25278220 PMC4401121

[2] Chalmers RM, Davies AP. Minireview: clinical cryptosporidiosis. Exp Parasitol. 2010;124(1):138–46. doi: 10.1016/j.exppara.2009.02.003 19545516

[3] Khalil IA, Troeger C, Rao PC, Blacker BF, Brown A, Brewer TG, et al. Morbidity, mortality, and long-term consequences associated with diarrhoea from Cryptosporidium infection in children younger than 5 years: a meta-analyses study. The Lancet Global Health. 2018;6(7):e758–68. doi: 10.1016/s2214-109x(18)30283-3 PMC6005120 29903377

[4] Zahedi A, Ryan U. Cryptosporidium - An update with an emphasis on foodborne and waterborne transmission. Res Vet Sci. 2020;132:500–12. doi: 10.1016/j.rvsc.2020.08.002 32805698

[5] Khan SM, Witola WH. Past, current, and potential treatments for cryptosporidiosis in humans and farm animals: A comprehensive review. Front Cell Infect Microbiol. 2023;13:1115522. doi: 10.3389/fcimb.2023.1115522 36761902 PMC9902888

[6] Ryan U, Zahedi A, Feng Y, Xiao L. An Update on Zoonotic Cryptosporidium Species and Genotypes in Humans. Animals (Basel). 2021;11(11):3307. doi: 10.3390/ani11113307 34828043 PMC8614385

[7] Guérin A, Roy NH, Kugler EM, Berry L, Burkhardt JK, Shin J-B, et al. Cryptosporidium rhoptry effector protein ROP1 injected during invasion targets the host cytoskeletal modulator LMO7. Cell Host Microbe. 2021;29(9):1407-1420.e5. doi: 10.1016/j.chom.2021.07.002 34348092 PMC8475647

[8] Guérin A, Striepen B. The Biology of the Intestinal Intracellular Parasite Cryptosporidium. Cell Host Microbe. 2020;28(4):509–15. doi: 10.1016/j.chom.2020.09.007 33031769

[9] Valigurová A, Jirků M, Koudela B, Gelnar M, Modrý D, Slapeta J. Cryptosporidia: epicellular parasites embraced by the host cell membrane. Int J Parasitol. 2008;38(8–9):913–22. doi: 10.1016/j.ijpara.2007.11.003 18158154

[10] Guérin A, Strelau KM, Barylyuk K, Wallbank BA, Berry L, Crook OM, et al. Cryptosporidium uses multiple distinct secretory organelles to interact with and modify its host cell. Cell Host Microbe. 2023;31(4):650-664.e6. doi: 10.1016/j.chom.2023.03.001 36958336

[11] Marino ND, Panas MW, Franco M, Theisen TC, Naor A, Rastogi S, et al. Identification of a novel protein complex essential for effector translocation across the parasitophorous vacuole membrane of Toxoplasma gondii. PLoS Pathog. 2018;14(1):e1006828. doi: 10.1371/journal.ppat.1006828 29357375 PMC5794187

[12] Seizova S, Ferrel A, Boothroyd J, Tonkin CJ. Toxoplasma protein export and effector function. Nat Microbiol. 2024;9(1):17–28. doi: 10.1038/s41564-023-01563-z 38172621

[13] Rosowski EE, Lu D, Julien L, Rodda L, Gaiser RA, Jensen KDC, et al. Strain-specific activation of the NF-kappaB pathway by GRA15, a novel Toxoplasma gondii dense granule protein. J Exp Med. 2011;208(1):195–212. doi: 10.1084/jem.20100717 21199955 PMC3023140

[14] Alaganan A, Fentress SJ, Tang K, Wang Q, Sibley LD. Toxoplasma GRA7 effector increases turnover of immunity-related GTPases and contributes to acute virulence in the mouse. Proc Natl Acad Sci U S A. 2014;111(3):1126–31. doi: 10.1073/pnas.1313501111 24390541 PMC3903209

[15] O’Hara SP, Yu J-R, Lin JJ-C. A novel Cryptosporidium parvum antigen, CP2, preferentially associates with membranous structures. Parasitol Res. 2004;92(4):317–27. doi: 10.1007/s00436-003-1057-5 14727189

[16] Zeng B, Cai X, Zhu G. Functional characterization of a fatty acyl-CoA-binding protein (ACBP) from the apicomplexan Cryptosporidium parvum. Microbiology (Reading). 2006;152(Pt 8):2355–63. doi: 10.1099/mic.0.28944-0 16849800 PMC1513434

[17] Zhang H, Guo F, Zhu G. Cryptosporidium Lactate Dehydrogenase Is Associated with the Parasitophorous Vacuole Membrane and Is a Potential Target for Developing Therapeutics. PLoS Pathog. 2015;11(11):e1005250. doi: 10.1371/journal.ppat.1005250 26562790 PMC4642935

[18] Guo F, Zhang H, Payne HR, Zhu G. Differential Gene Expression and Protein Localization of Cryptosporidium parvum Fatty Acyl-CoA Synthetase Isoforms. J Eukaryot Microbiol. 2016;63(2):233–46. doi: 10.1111/jeu.12272 26411755 PMC4775295

[19] He W, Sun L, Hou T, Yang Z, Yang F, Zhang S, et al. SKSR1 identified as key virulence factor in Cryptosporidium by genetic crossing. Nat Commun. 2025;16(1):4694. doi: 10.1038/s41467-025-60088-7 40394032 PMC12092579

[20] Li M, Yang F, Hou T, Gong X, Li N, Sibley LD, et al. Variant surface protein GP60 contributes to host infectivity of Cryptosporidium parvum. Commun Biol. 2024;7(1):1175. doi: 10.1038/s42003-024-06885-0 39294220 PMC11411101

[21] Shaw S, Li X, Buenconsejo GY, Zhou TH, Cohen A, Yasur-Landau D, et al. Genetic crosses reveal genomic loci responsible for virulence in Cryptosporidium parvum infection. Cell Rep. 2025;44(10):116315. doi: 10.1016/j.celrep.2025.116315 40971299 PMC12590416

[22] Walzer KA, Tandel J, Byerly JH, Daniels AM, Gullicksrud JA, Whelan EC, et al. Transcriptional control of the Cryptosporidium life cycle. Nature. 2024;630(8015):174–80. doi: 10.1038/s41586-024-07466-1 38811723 PMC12057246

[23] Rodrigues E, Pallett MA, Straker LC, Mkandawire TT, Sala K, Collinson L, et al. Cryptosporidium modifies intestinal microvilli through an exported virulence factor. Cell Host Microbe. 2025;33(5):719-730.e5. doi: 10.1016/j.chom.2025.04.001 40300595 PMC7618952

[24] Lopez J, Bittame A, Massera C, Vasseur V, Effantin G, Valat A, et al. Intravacuolar Membranes Regulate CD8 T Cell Recognition of Membrane-Bound Toxoplasma gondii Protective Antigen. Cell Reports. 2015;13(10):2273–86. doi: 10.1016/j.celrep.2015.11.001 26628378

[25] Griffith MB, Pearce CS, Heaslip AT. Dense granule biogenesis, secretion, and function in Toxoplasma gondii. J Eukaryot Microbiol. 2022;69(6):e12904. doi: 10.1111/jeu.12904 35302693 PMC9482668

[26] Melicherová J, Hofmannová L, Valigurová A. Response of cell lines to actual and simulated inoculation with Cryptosporidium proliferans. Eur J Protistol. 2018;62:101–21. doi: 10.1016/j.ejop.2017.12.003 29316479

[27] Mayoral J, Guevara RB, Rivera-Cuevas Y, Tu V, Tomita T, Romano JD, et al. Dense Granule Protein GRA64 Interacts with Host Cell ESCRT Proteins during Toxoplasma gondii Infection. mBio. 2022;13(4):e0144222. doi: 10.1128/mbio.01442-22 35730903 PMC9426488

[28] Beck JR, Ho C-M. Transport mechanisms at the malaria parasite-host cell interface. PLoS Pathog. 2021;17(4):e1009394. doi: 10.1371/journal.ppat.1009394 33793667 PMC8016102

[29] Cygan AM, Jean Beltran PM, Mendoza AG, Branon TC, Ting AY, Carr SA, et al. Proximity-Labeling Reveals Novel Host and Parasite Proteins at the Toxoplasma Parasitophorous Vacuole Membrane. mBio. 2021;12(6):e0026021. doi: 10.1128/mBio.00260-21 34749525 PMC8576527

[30] Abrahamsen MS, Templeton TJ, Enomoto S, Abrahante JE, Zhu G, Lancto CA, et al. Complete genome sequence of the apicomplexan, Cryptosporidium parvum. Science. 2004;304(5669):441–5. doi: 10.1126/science.1094786 15044751

[31] Xu Z, Guo Y, Roellig DM, Feng Y, Xiao L. Comparative analysis reveals conservation in genome organization among intestinal Cryptosporidium species and sequence divergence in potential secreted pathogenesis determinants among major human-infecting species. BMC Genomics. 2019;20(1):406. doi: 10.1186/s12864-019-5788-9 31117941 PMC6532270

[32] Guo Y, Tang K, Rowe LA, Li N, Roellig DM, Knipe K, et al. Comparative genomic analysis reveals occurrence of genetic recombination in virulent Cryptosporidium hominis subtypes and telomeric gene duplications in Cryptosporidium parvum. BMC Genomics. 2015;16(1):320. doi: 10.1186/s12864-015-1517-1 25903370 PMC4407392

[33] Nader JL, Mathers TC, Ward BJ, Pachebat JA, Swain MT, Robinson G, et al. Evolutionary genomics of anthroponosis in Cryptosporidium. Nat Microbiol. 2019;4(5):826–36. doi: 10.1038/s41564-019-0377-x 30833731

[34] Dubremetz JF, Achbarou A, Bermudes D, Joiner KA. Kinetics and pattern of organelle exocytosis duringToxoplasma gondii/host-cell interaction. Parasitol Res. 1993;79(5):402–8. doi: 10.1007/bf00931830 8415546

[35] Dunn JD, Ravindran S, Kim S-K, Boothroyd JC. The Toxoplasma gondii dense granule protein GRA7 is phosphorylated upon invasion and forms an unexpected association with the rhoptry proteins ROP2 and ROP4. Infect Immun. 2008;76(12):5853–61. doi: 10.1128/IAI.01667-07 18809661 PMC2583583

[36] Grützke J, Rindte K, Goosmann C, Silvie O, Rauch C, Heuer D, et al. The spatiotemporal dynamics and membranous features of the Plasmodium liver stage tubovesicular network. Traffic. 2014;15(4):362–82. doi: 10.1111/tra.12151 24423236

[37] Ingmundson A, Alano P, Matuschewski K, Silvestrini F. Feeling at home from arrival to departure: protein export and host cell remodelling during Plasmodium liver stage and gametocyte maturation. Cell Microbiol. 2014;16(3):324–33. doi: 10.1111/cmi.12251 24330249

[38] Sylvester K, Maher SP, Posfai D, Tran MK, Crawford MC, Vantaux A, et al. Characterization of the Tubovesicular Network in Plasmodium vivax Liver Stage Hypnozoites and Schizonts. Front Cell Infect Microbiol. 2021;11:687019. doi: 10.3389/fcimb.2021.687019 34195101 PMC8236947

[39] Anonymous. Guide for the Care and Use of Laboratory Animals. 8th ed. Animals NRCUCftUotGftCaUoL editor, editor. Washington (DC): National Academies Press (US); 2011.

[40] Li N, Zhao W, Song S, Ye H, Chu W, Guo Y, et al. Diarrhoea outbreak caused by coinfections of Cryptosporidium parvum subtype IIdA20G1 and rotavirus in pre-weaned dairy calves. Transbound Emerg Dis. 2022;69(5):e1606–17. doi: 10.1111/tbed.14496 35226796

[41] Jia R, Huang W, Huang N, Yu Z, Li N, Xiao L, et al. High infectivity and unique genomic sequence characteristics of Cryptosporidium parvum in China. PLoS Negl Trop Dis. 2022;16(8):e0010714. doi: 10.1371/journal.pntd.0010714 PMC9436107 35994488

[42] Arrowood MJ, Donaldson K. Improved purification methods for calf-derived Cryptosporidium parvum oocysts using discontinuous sucrose and cesium chloride gradients. J Eukaryot Microbiol. 1996;43(5):89S. doi: 10.1111/j.1550-7408.1996.tb05015.x 8822880

[43] Alves M, Xiao L, Sulaiman I, Lal AA, Matos O, Antunes F. Subgenotype Analysis of Cryptosporidium Isolates from Humans, Cattle, and Zoo Ruminants in Portugal. J Clin Microbiol. 2003;41(6):2744–7. doi: 10.1128/jcm.41.6.2744-2747.2003 12791920 PMC156540

[44] Almagro Armenteros JJ, Tsirigos KD, Sønderby CK, Petersen TN, Winther O, Brunak S, et al. SignalP 5.0 improves signal peptide predictions using deep neural networks. Nat Biotechnol. 2019;37(4):420–3. doi: 10.1038/s41587-019-0036-z 30778233

[45] Sigrist CJA, de Castro E, Cerutti L, Cuche BA, Hulo N, Bridge A, et al. New and continuing developments at PROSITE. Nucleic Acids Res. 2013;41(Database issue):D344-7. doi: 10.1093/nar/gks1067 23161676 PMC3531220

[46] Gasteiger E, Hoogland C, Gattiker A, Duvaud S, Wilkins MR, Appel RD, et al. Protein Identification and Analysis Tools on the ExPASy Server. In: Walker JM, editor. The Proteomics Protocols Handbook. Totowa, NJ: Humana Press; 2005. p. 571–607. doi: 10.1385/1-59259-890-0:571

[47] Romero P, Obradovic Z, Li X, Garner EC, Brown CJ, Dunker AK. Sequence complexity of disordered protein. Proteins. 2000;42(1):38–48. doi: 10.1002/1097-0134(20010101)42:1<38::aid-prot50>3.0.co;2-3 11093259

[48] Steentoft C, Vakhrushev SY, Joshi HJ, Kong Y, Vester-Christensen MB, Schjoldager KT-BG, et al. Precision mapping of the human O-GalNAc glycoproteome through SimpleCell technology. EMBO J. 2013;32(10):1478–88. doi: 10.1038/emboj.2013.79 23584533 PMC3655468

[49] Sateriale A, Pawlowic M, Vinayak S, Brooks C, Striepen B. Genetic Manipulation of *Cryptosporidium parvum* with CRISPR/Cas9. Method Mol Biol. 2020;2052:219–28. doi: 10.1007/978-1-4939-9748-0_13 31452165

[50] Vinayak S, Pawlowic MC, Sateriale A, Brooks CF, Studstill CJ, Bar-Peled Y, et al. Genetic modification of the diarrhoeal pathogen Cryptosporidium parvum. Nature. 2015;523(7561):477–80. doi: 10.1038/nature14651 26176919 PMC4640681

[51] Xu R, Feng Y, Xiao L, Sibley LD. Insulinase-like Protease 1 Contributes to Macrogamont Formation in Cryptosporidium parvum. mBio. 2021;12(2):e03405-20. doi: 10.1128/mBio.03405-20 33688009 PMC8092296

[52] Pawlowic MC, Vinayak S, Sateriale A, Brooks CF, Striepen B. Generating and Maintaining Transgenic Cryptosporidium parvum Parasites. Curr Protoc Microbiol. 2017;46:20B.2.1-20B.2.32. doi: 10.1002/cpmc.33 28800157 PMC5556942

[53] Yu X, Guo F, Mouneimne RB, Zhu G. Cryptosporidium parvum Elongation Factor 1α Participates in the Formation of Base Structure at the Infection Site During Invasion. J Infect Dis. 2020;221(11):1816–25. doi: 10.1093/infdis/jiz684 31872225 PMC7213558

[54] Dos Santos Pacheco N, Soldati-Favre D. Coupling auxin-inducible degron system with ultrastructure expansion microscopy to accelerate the discovery of gene function in Toxoplasma gondii. In: de Pablos LM, Sotillo J, editors. Parasite Genomics: Methods and Protocols. New York, NY: Springer US; 2021. p. 121–37. 10.1007/978-1-0716-1681-9_8 34313987

[55] Gambarotto D, Zwettler FU, Le Guennec M, Schmidt-Cernohorska M, Fortun D, Borgers S, et al. Imaging cellular ultrastructures using expansion microscopy (U-ExM). Nat Methods. 2019;16(1):71–4. doi: 10.1038/s41592-018-0238-1 30559430 PMC6314451

[56] Deng M, Hou T, Zhang J, Mao X, Yang F, Wei Y, et al. Cultivation, cryopreservation, and transcriptomic studies of host-adapted Cryptosporidium parvum and Cryptosporidium hominis using enteroids. iScience. 2024;27(4):109563. doi: 10.1016/j.isci.2024.109563 38623332 PMC11016910

